# Task‐Specific Personalized Theta tACS Modulates Theta Dynamics in Associative Memory

**DOI:** 10.1002/brb3.71259

**Published:** 2026-02-16

**Authors:** Busra Nur Kahraman, Mevhibe Saricaoglu, Lutfu Hanoglu

**Affiliations:** ^1^ Research Institute for Health Sciences and Technologies (SABITA), Regenerative and Restorative Medicine Research Center (REMER), Clinical Electrophysiology, Neuroimaging and Neuromodulation Lab Istanbul Medipol University Istanbul Turkey; ^2^ Vocational School, Program of Electroneurophysiology Istanbul Medipol University Istanbul Turkey; ^3^ Department of Neurology Istanbul Medipol University Istanbul Turkey

**Keywords:** associative memory, EEG, individual theta frequency, tACS, theta oscillation

## Abstract

**Purpose:**

Associative memory is fundamental to human cognition and has been strongly linked to neural oscillations in the theta frequency band. Rather than being confined to a single brain region, these processes are thought to emerge from dynamic interactions among temporal, frontal, and parietal areas, as proposed by the Temporo–Frontal–Parietal Network Model. The role of the parietal cortex plays a central and dynamic role in associative memory by supporting integrative processes that enable successful retrieval. The present study investigated whether transcranial alternating current stimulation (tACS) delivered on the left parietal cortex at individualized theta frequency (ITF) could modulate associative memory performance.

**Method:**

Thirty healthy participants were randomly assigned to either a stimulation or sham condition. During the encoding phase of the Face and Scene Task (FAST), EEG recordings were collected. Each participant's ITF, derived from their theta activity during the encoding task, was calculated. ITF tACS was delivered over the P3 site to the stimulation group, whereas the sham group received sham stimulation. Following encoding, all participants completed a recognition task. EEG, behavioral performance, and theta activity were compared across groups.

**Finding:**

The tACS group did not differ in recognition performance from those in the sham group. No significant effects were observed on spontaneous EEG with eyes open or directly on ITF. Time–frequency analyses revealed right‐hemispheric dominance in the stimulation group and left‐hemispheric dominance in the sham group within 100–400 ms. Comparisons between encoding and recognition phases suggested that stimulation modulated theta dynamics, contributing to hemispheric asymmetries.

**Conclusion:**

The tACS at ITF can change associative memory performance, although its effects on theta activity vary across spatial and temporal dimensions. The findings suggest that ITF tACS administered during encoding is associated with improved recognition performance compared to sham, highlighting the potential of personalized stimulation approaches to support memory processing.

## Graphical abstract

Left parietal theta‐tACS alters encoding‐related brain activity, hinting at enhanced neural efficiency, while recognition performance remains largely unchanged.

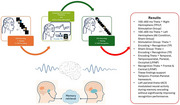

## 1 Introductıon

Associative memory refers to the process by which independent or seemingly unrelated pieces of information (e.g., face–name, object–location, event–place) are bound together through contextual integration, stored as a unified representation, and retrieved when needed (Staresina 2006; Suzuki 2007). Such associations, whether within the same modality (e.g., visual: object–location) or across modalities (e.g., visual–verbal: face–name), enable individuals to recall interconnected experiences or knowledge (Becker et al. 2015; Bjekić et al. 2023). At its core, associative memory involves the formation and maintenance of relational links that allow discrete items of information to be later recalled together.

The neural basis of associative memory relies on a multicomponent process supported by medial temporal lobe structures and a broader temporo–frontal–parietal network (Hermiller et al. 2019). Central to this network is the hippocampus, which binds information from different sensory and cognitive modalities to form coherent associative representations (Davachi 2006; Eichenbaum 2017). Hippocampal functions are not limited to the medial temporal lobe; they are also supported through strong connections with the posterior parietal cortex (PPC) (Kota et al. 2020). While the hippocampus is critical for relational binding, parietal regions regulate aspects of attention, reliability, and contextual richness in these representations (Kim 2011; Staresina 2006; Sun et al. 2025).

The parietal cortex plays a central and dynamic role in associative memory by supporting the attentional and integrative processes that enable successful retrieval (Cabeza et al. 2011; Levy 2012). Rather than serving as a passive storage area, it actively regulates how stored associations are accessed and reconstructed (Shimamura 2011). Through its extensive connections with medial temporal and prefrontal regions, the parietal cortex functions as a hub that links attentional control, memory retrieval, and the reconstruction of past experiences (Sestieri et al. 2011). This integration allows the parietal cortex to transform discrete memory traces into unified, context‐rich representations that support associative remembering (Hayes et al. 2011). Beyond theoretical accounts, electrophysiological studies have consistently linked theta oscillations (4–7 Hz)—particularly measured in fronto–parietal regions—to memory processes, including associative memory (Herweg et al. 2020). Cortical theta oscillations are thought to reflect communication with the hippocampus, proposed as a key driver of memory functions, and to support synchronized information transfer across brain regions. In doing so, theta oscillations play a critical role in encoding, integration, consolidation from short‐ to long‐term memory, and retrieval (Aktürk et al. 2022; Clouter et al. 2017). Accordingly, theta frequency has been recognized as a central electrophysiological marker of memory function.

To investigate the causal influence of theta frequency on cognition, transcranial alternating current stimulation (tACS) has been widely employed in recent years (Klink et al. 2020; Staudigl and Hanslmayr 2013). By modulating endogenous brain rhythms, tACS enables direct testing of oscillatory contributions to cognitive processes (Antal and Paulus 2013). The technique applies low‐intensity alternating current at a target frequency to stimulate relevant brain networks (Antal and Paulus 2013). Studies using theta‐frequency tACS have demonstrated that stimulation targeting the PPC can enhance associative memory performance, although some studies have also reported reduced or non‐significant effects under certain stimulation parameters or task conditions (Meng et al. 2021; Sun et al. 2025; Živanović et al. 2022).

However, interindividual variability and stimulation parameters have been identified as key factors underlying inconsistent findings in the literature. Fixed‐frequency or non‐individualized stimulation may fail to support—and may even disrupt—endogenous theta rhythms. For this reason, current research increasingly emphasizes personalized tACS protocols, in which individual theta frequency (ITF) is measured during task performance and used to guide stimulation (Bjekić et al. 2022; Lang et al. 2019).

The aim of this study was to investigate the effect of task‐specific stimulation at the ITF on associative memory processes and to evaluate electrophysiological changes in theta oscillations as a potential neural correlate of these effects. Given that the PPC has been validated as a key target region for the modulation of associative memory and that theta entrainment during encoding may represent a plausible underlying mechanism, this study tested whether ITF‐tACS applied over the P3 during encoding enhances subsequent recognition accuracy.

## 2 Materials and Methods

### 2.1 Participants

A total of 30 healthy participants aged between 18 and 30 years were included in the study. The required sample size was determined using G*Power 3.1. The power analysis—conducted based on the three measurements obtained from the associative memory recognition phase (Old Pairs, Recombined Pairs, and New Pairs) and using two groups for comparison—indicated that a total sample of 30 participants would be adequate to detect a large effect size (f = 0.83) with a 5% significance level (α = 0.05) and 83% statistical power (1 − β = 0.83). (Cohen, 1988). Inclusion criteria for healthy participants were as follows: no history of neurological or psychiatric diagnosis and no use of medication; right‐handedness; a minimum of 12 years of formal education; and performance within the normal range on the Montreal Cognitive Assessment (MoCA) (Ozdilek and Kenangil 2014). All participants had normal vision. None of the participants had prior experience with electrical brain stimulation, nor had they previously performed the cognitive tasks included in the experimental protocol. Written informed consent was obtained from all participants prior to participation, in accordance with the approval of the Local Ethics Committee of Istanbul Medipol University (Ethics Report No: E‐10840098‐772.02‐6571). Participants were randomly assigned to one of two groups: stimulation or sham.

### 2.2 Experimental Design and Procedure

The study employed a randomized, sham‐controlled, single‐blind parallel group design. All procedures were conducted within a single session for each participant. In this study, the Face and Scene Task (FAST) was used for evaluating associative memory.

**FIGURE 1:**
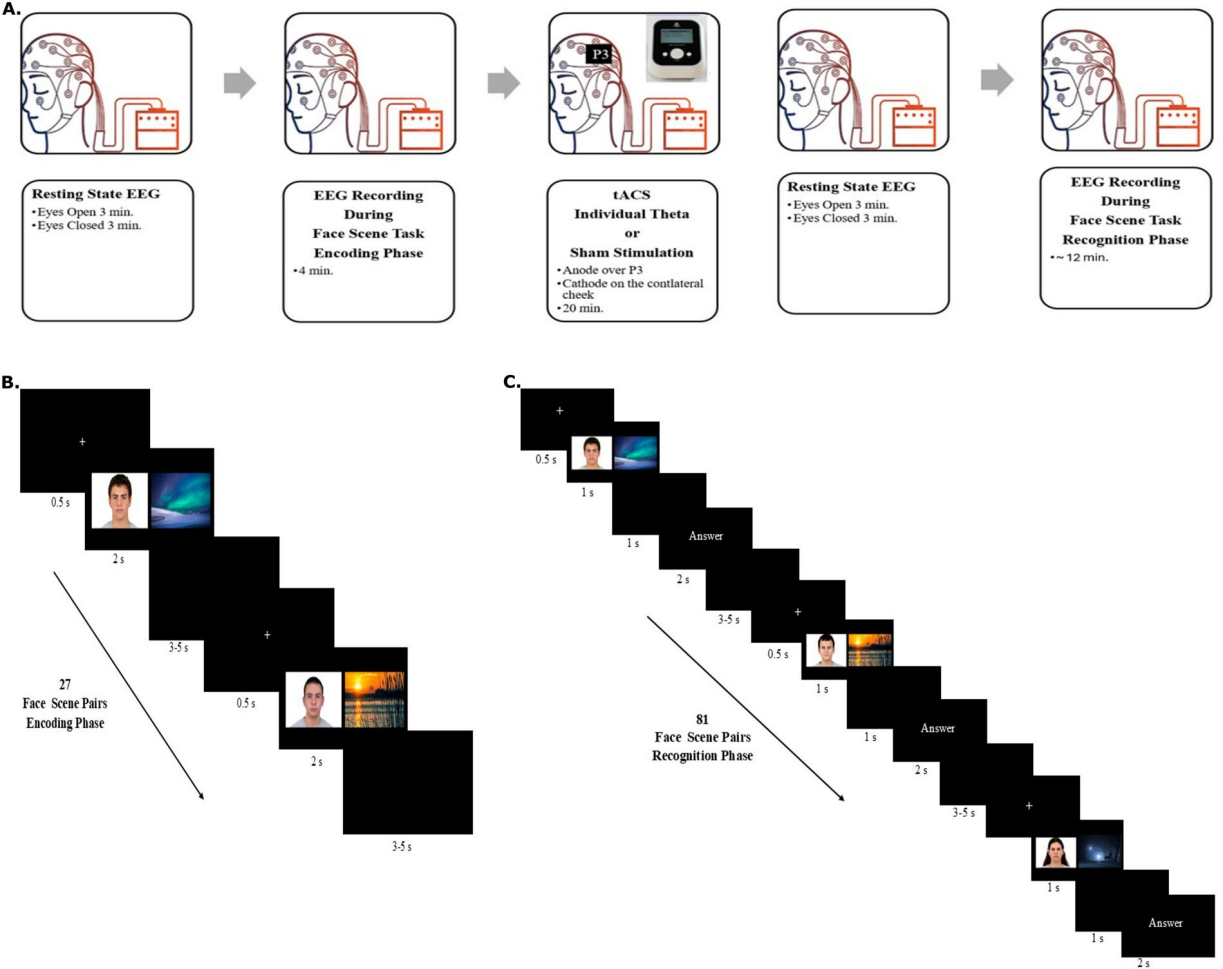
**Experimental design and task procedure**. (A) Experimental design, (B) Face scene task encoding phase, and (**C)** Face scene task recognition phase (**Abbreviations**: min, minutes; s, seconds).

The experiment consisted of five consecutive phases (Figure 1A). First, a baseline resting‐state EEG recording was obtained for 3 min while participants remained seated with their eyes open and relaxed. Next, participants performed the FAST encoding phase, during which EEG data were continuously recorded to capture neural activity associated with associative encoding. After the encoding phase was completed, EEG recording was paused, and each participant's ITF was calculated based on the EEG data collected during encoding. This calculation took approximately two minutes for each participant. To avoid temporal bias and to ensure equal time elapsed, calculations were performed for both the sham and stimulation groups. Following this, participants received either sham stimulation or tACS stimulation in their respective ITF, depending on their group assignment. No EEG recording was conducted during the stimulation period. For the stimulation phase, the EEG electrode at P3 was temporarily removed, and the tACS anode was positioned underneath the EEG cap in its place. In the stimulation group, tACS was applied over the left posterior parietal cortex (anode: P3; cathode: contralateral cheek) at each participant's calculated individual theta frequency, delivered at 2 mA for 20 min (Živanović et al. 2022). In the sham group, stimulation was applied with identical electrode placement at 2 mA for 20 min but followed a sham protocol. Upon completion of the stimulation, the EEG electrode was reinstalled at the P3 site for subsequent recordings. Immediately after stimulation, a 3‐min eyes‐open resting EEG recording was taken under the same conditions as at baseline. Finally, participants completed the FAST recognition phase, during which EEG data were again recorded to examine neural dynamics associated with associative retrieval.

### 2.3 FAST Task

The FAST is an associative memory task that measures participants' ability to learn and later recall information by viewing both face and scene images simultaneously. In the task, a human face image is typically paired with a scene (landscape) image. During the encoding phase, the participant learns this pairing, and then, during the recognition phase, attempts to distinguish whether the face‐scene pair is a previously seen pair or a newly combined pair. This task is frequently used to assess associative memory, recognition memory, and parietal memory (Lang et al. 2019).

During the encoding phase of the FAST, 27 face–scene pairs were presented in randomized order. Each trial began with a white fixation cross displayed on a black background for 500 ms. The stimulus pair was then presented for 2 s to allow encoding. Following stimulus presentation, a black screen was shown during a jittered interstimulus interval of 3–5 s. Participants were instructed to encode the pairs with the intention of later remembering the face and scene together, but no additional task demands were given (Figure 1B).

The FAST recognition phase consisted of three types of stimuli presented in randomized order: (Aktürk et al. 2022) 27 Old Pairs (OP), identical face–scene pairs shown during encoding; (Ali et al. 2013) 27 Recombined Pairs (RP), consisting of faces and scenes previously seen during encoding but recombined into novel pairings; and (Antal and Paulus 2013) 27 New Pairs (NP), composed of entirely new faces and scenes not presented before. In total, 81 pairs were presented. Participants were instructed, “Look carefully at the image. If you saw both pictures together during encoding (OP), press 1; if you saw both pictures but not together (RP), press 2; if you have not seen either picture before (NP), press 3.*”* Each trial began with a 500 ms fixation cross, followed by stimulus presentation for 1 s. A black screen was then shown for 1 s, after which the instruction “Respond now” appeared, prompting participants to provide their response. Following the response, a black screen was presented during a jittered interstimulus interval of 3–5 s (Figure 1C). The task was implemented following the procedure described by Lang et al. While we did not use the exact same images, we selected stimuli from the links provided in their publication, and the classification procedure was conducted as specified by Lang et al. (2019). During encoding, participants were presented with pairs of neutral faces and natural landscapes displayed side by side as a single image. Neutral faces of diverse age groups and ethnic backgrounds were selected from the Chicago Face Database (CFD Version 2.0.3; Ma et al. 2015; https://www.chicagofaces.org/). Faces did not include distinctive features such as tattoos or piercings. Factors such as ethnicity/age were chosen to be controlled and balanced. Landscape images were obtained from an open‐access database (www.pixabay.com) (Meng et al. 2021).

The experimental design and stimulus presentation were implemented in E‐Prime software (Psychology Software Tools Inc., Pittsburgh, PA). All stimuli were displayed in full‐screen mode on a monitor measuring 47.5 × 26.8 cm, with a refresh rate of 60 Hz, positioned at a viewing distance of 90 cm. Stimuli subtended approximately 19° horizontally and 16° vertically.

### 2.4 tACS Protocol

tACS was delivered during the resting state using a sinusoidal waveform at 2 mA peak‐to‐peak (0‐2 mA) for 20 min with a Neuromars device (Marslabs, Turkey). The stimulation included a 30‐s ramp‐up from 0 to +2 mA and 60 s stay at 2 mA, and a 30‐s ramp‐down at the end. In the sham condition, the same procedure was used except that after the 30‐second ramp‐up, only 60 s of stimulation at 0–2 mA were delivered, followed by a 30‐second ramp‐down to 0 mA, with no current for the remainder of the 20‐min session.

Before stimulation, scalp sites were cleaned with alcohol wipes to reduce impedance. Electrode placement sites were marked on the scalp using an EEG cap, and stimulation electrodes were positioned directly beneath the marked areas after baseline EEG recording. Two saline‐soaked round sponge electrodes (5‐cm diameter) were used. Sponge electrode impedances were kept below 10 kΩ throughout the procedure.

To modulate the left posterior parietal cortex—shown to play a critical role in associative memory—the anode was positioned over P3. The cathode was placed on the contralateral cheek to avoid physiologically meaningful changes in cortical regions unrelated to the task (Bjekić et al. 2019; Vulić et al. 2021).

For participants in the stimulation group, the stimulation frequency was determined based on ITF. The ITF was calculated using Brain Vision Analyzer (BVA) software (BrainVision LLC, Morrisville, North Carolina, USA) from EEG data collected during the FAST encoding phase. The analysis procedure was as follows: EEG data were segmented with the onset of the visual stimulus defined as time zero; all visual pairs presented during coding were used; and epochs from –2000 to +2000 ms were extracted. Segments were baseline‐corrected using the post‐stimulus 2000 ms window, and artifacts (e.g., eye movements, muscle activity) were removed. Cleaned and segmented data were then subjected to Fast Fourier Transform (FFT) (Hanning window, zero‐padding, 0.1 Hz frequency resolution) to obtain power spectra for each segment, which were subsequently averaged.

The ITF was identified as the frequency within the theta band (4–7 Hz) showing maximum power across the average spectrum of signals recorded from the C3, Cz, C4, P3, Pz, and P4 electrodes, which overlie regions previously implicated in associative memory (Bjekić et al., 2022). The frequency corresponding to the maximum theta power was consistently selected as the ITF for stimulation.

### 2.5 EEG Recording

EEG data were recorded using Brain Vision Recorder software (Brain Products, Munich, Germany). A 32‐channel EEG cap (actiCAP 128, Brain Products GmbH, Germany) was employed, with electrodes placed according to the international 10–20 system. Recordings were obtained from the following electrode sites: FP1, FP2, F7, F3, Fz, F4, F8, FT7, FC3, FCz, FC4, FT8, T7, C3, Cz, C4, T8, TP7, CP3, CPz, CP4, TP8, P7, P3, Pz, P4, P8, O1, Oz, and O2. The ground electrode was positioned behind the right earlobe, and the reference electrode was placed in front of it.

To monitor ocular artifacts, a horizontal electrooculogram (EOGH) electrode was positioned below the left eye, and a vertical electrooculogram (EOGV) electrode was placed above the same eye. Before EEG recording, the scalp was prepared to ensure optimal signal quality and low electrode impedance. The skin at each electrode site was gently cleaned with an alcohol wipe to remove oil and surface debris. A small amount of mild abrasive gel (Nuprep) was applied to further reduce impedance. EEG electrodes were then attached using conductive gel (for gel‐based caps). Electrode impedance was checked prior to data collection and maintained below 5 kΩ across all channels to ensure stable signal transmission. These steps minimized noise and cross‐contamination between sessions and ensured consistent electrode performance. Before the tACS stimulation, the EEG electrode at P3 was removed, and the site was cleaned in accordance with the tACS protocol to prepare for anode placement. After stimulation, the P3 electrode was reattached using conductive gel. The impedance levels were maintained below 5 kΩ for the ground and reference electrodes and below 10 kΩ for scalp electrodes. EEG signals were digitized online at a sampling rate of 500 Hz, with a band‐pass filter of 0.01–250 Hz. All recordings took place in a dimly lit, sound‐attenuated laboratory, with participants seated comfortably.

### 2.6 EEG Data Analysis

EEG data were analyzed using Brain Vision Analyzer 2.2.

For spontaneous EEG, the eyes‐open condition was analysed. A broad filter (0.01 Hz high‐pass and 50 Hz low‐pass) was applied to remove low‐ and high‐frequency noise. Raw data were segmented into 1‐s epochs, and epochs contaminated by ocular, blink, or muscle artifacts were manually removed offline by a senior EEG researcher. Subsequently, FFT (0.9 Hz maximum resolution, 10% Hanning window) was applied to obtain the power spectrum. The average FFT power spectrum was calculated across epochs. Since stimulation was applied at each participant's ITF, the analysis focused on the theta band (4–7 Hz). Peak theta amplitude values were extracted, and the highest theta peak for each participant was used in statistical analyses.

For calculating spontaneous ITF, global power spectra were derived from eyes‐open resting‐state EEG data. FFT (Hanning window, zero‐padding, 0.1 Hz frequency resolution) was applied to preprocessed data, and spectra were averaged across epochs. ITF was defined as the maximum theta power within 4–7 Hz at the electrode cluster (C3, Cz, C4, P3, Pz, P4), previously linked to associative memory (Bjekić et al. 2022).

For event‐related ITF analysis, EEG data were obtained during the FAST (encoding and recognition phases), using only correctly answered trials (OP, RP, NP separately). Segmentation was performed by setting the onset of visual stimuli as time zero and extracting epochs from –2000 to +2000 ms. Segments were baseline‐corrected using the 1000 ms post‐stimulus window, and artifacts (ocular, muscle) were removed. Cleaned segments were analyzed using FFT (Hanning window, zero‐padding, 0.1 Hz frequency resolution), and averaged spectra were calculated. Event‐related ITF was defined as the frequency with maximum theta power (4–7 Hz) at the electrode cluster (C3, Cz, C4, P3, Pz, P4), consistent with prior associative memory research (Bjekić et al. 2022).

For event‐related spectral analyses, data were filtered (0.01–50 Hz), and ocular artifacts were removed using Independent Component Analysis (ICA). Segmentation was performed according to stimulus type (OP, RP, NP) and response accuracy. Only correct responses were included. Stimulus‐locked epochs were extracted (−4000 to +4000 ms relative to stimulus onset), and further segmentation (−2000 to +2000 ms) was applied to remove blink‐ and motor‐related noise.

Event‐related power analysis was conducted using a complex Morlet Wavelet Transform with 3‐cycle wavelet widths in the theta (4–7 Hz). The theta band was subdivided into 60 logarithmically scaled bins. Wavelet Transform (WT) was computed, and event‐related power was calculated by averaging single‐trial WT results. Post‐stimulus responses were normalized against a pre‐stimulus baseline (−500 to −300 ms) and converted into decibels (dB). Numerical values for theta band power were exported within the 100–400 ms window, with frequency layers 1–60 included. Point mean normalization was applied to all exported wavelet data. The 100–400  ms theta response was analyzed because this time window has been shown to be critical for memory processing and because the data exhibited pronounced theta power specifically within this interval (White et al. 2013; Solomon et al. 2019).

Only correct responses were included in the EEG analyses. This decision was theoretically motivated by the aim of the study, which was to examine theta activity associated specifically with successful encoding and accurate retrieval. Incorrect or guessed responses do not necessarily reflect genuine retrieval processes and may involve qualitatively different mechanisms such as guessing, fabrication, or retrieval failure. Including these trials would therefore introduce noise and potentially obscure the neural activity linked to successful memory processing. In addition, the number of incorrect or omitted trials varied considerably across participants and was often insufficient for reliable condition‐wise analysis. For these reasons, restricting the analysis to correct trials provided the most valid and stable basis for investigating the neural correlates of successful memory performance. In addition, for each EEG segment, care was taken to ensure comparable numbers of segments both within each participant's tasks and across participants, thereby minimizing potential biases.

### 2.7 Statistical Analysis

Statistical analyses were conducted using IBM SPSS Statistics 22 (IBM Corp., Armonk, NY, USA) and Jamovi (The Jamovi project, 2021). Descriptive statistics are presented as mean ± standard deviation. The normality of data distribution was assessed using the Kolmogorov–Smirnov test. Independent samples *t*‐tests were used to compare demographic variables and MoCA scores.

During the FAST recognition phase, group differences in correct and incorrect responses for OP, RP, and NP stimuli were assessed using independent samples t‐tests. This analysis approach was retained to ensure methodological consistency and direct comparability with previous tACS–memory studies. To further validate the findings and increase analytical rigor, recognition performance was additionally examined using signal detection theory (SDT) and a generalized linear mixed‐effects model (GLMM). These complementary analyses were conducted to confirm whether more sensitive modeling approaches would reveal effects not captured by classical group‐level tests. However, neither the SDT nor the GLMM analyses yielded meaningful or statistically significant effects; therefore, for clarity and focus, only the t‐test results are reported in the manuscript. EEG data were analyzed using ANOVA to compare groups. For spontaneous EEG analysis, only eyes‐open resting‐state EEG data were considered. Group (stimulation vs. sham) was included as a between‐subject factor, while time (pre‐stimulation vs. post‐stimulation), location (8 electrode clusters: Frontal 1 (F3 & F4), Frontal 2 (F7 & F8), Central, Temporal, Temporo‐Parietal, Parietal 1 (P3 & P4), Parietal 2 (P7 & P8), and Occipital), and hemisphere (left, right) were treated as within‐subject factors.

ANOVA was also applied to compare ITF values between the eyes‐open resting state (pre‐ and post‐stimulation) and the FAST encoding and recognition phases (correct responses for OP, RP, and NP stimuli). In these analyses, group (stimulation vs. sham) was treated as a between‐subject factor, and condition (pre‐stimulation, post‐stimulation, encoding, and correct OP, RP, and NP responses) was treated as a within‐subject factor.

For event‐related EEG analyses, group (stimulation vs. sham) was considered a between‐subject factor, while condition (theta responses for correct OP, RP, and NP trials), location (8 electrode clusters: Frontal 1 (F3 & F4), Frontal 2 (F7 & F8), Central, Temporal, Temporo‐Parietal, Parietal 1 (P3 & P4), Parietal 2 (P7 & P8), Occipital), and hemisphere (left, right) were included as within‐subject factors.

To examine differences between theta responses during encoding and each subsequent condition, a two‐condition mixed‐design repeated measures ANOVA was conducted. In this design, group (stimulation vs. sham) was included as a between‐subject factor, while condition (two conditions: encoding vs. correct OP theta response; encoding vs. correct RP theta response; encoding vs. correct NP theta response), location (8 electrode clusters: Frontal 1 (F3 & F4), Frontal 2 (F7 & F8), Central (C3 & C4), Temporal (T7 & T8), Temporo‐Parietal (TP7 & TP8), Parietal 1 (P3 & P4), Parietal 2 (P7 & P8), Occipital (O1 & O2)), and hemisphere (left, right) were treated as within‐subject factors.

Statistical significance was determined using Greenhouse‐Geisser corrected *p*‐values, and a threshold of *p* < 0.05 was applied for all tests.

## 3 Results

### 3.1 Demographic Data and Behavioral Outcomes

The study was conducted with a total of 30 healthy volunteers. Participants were randomly assigned to either the stimulation or sham group, with 15 individuals in each group.

Demographic variables were compared between groups using independent samples t‐tests. The analysis revealed no statistically significant differences between the groups at baseline, indicating that no evidence of baseline imbalance was detected (*p* > 0.05; Table 1). Across all analytical approaches, no significant differences were observed between the tACS and sham groups. First, independent samples t‐tests comparing correct and incorrect response counts for OP, RP, and NP stimuli revealed no significant group differences (all *p* > 0.05, Table 1). Similarly, the signal detection analysis showed that d′ (discriminability) did not differ between groups (*p* > 0.05), indicating comparable recognition sensitivity in the presence of RP stimuli. Finally, the trial‐level generalized linear mixed‐effects model (GLMM) with a logistic link function (Accuracy ∼ Group × StimulusType + (1 + StimulusType | Subject) + (1 | Item)) also yielded no significant main effect of Group, nor a Group × StimulusType interaction (all *p* > 0.05). Taken together, the classical t‐tests, SDT‐based metrics, and GLMM converged on the same outcome, demonstrating that tACS did not produce a measurable effect on recognition performance.

**TABLE 1:** Demographic and behavioral data of the groups.

	Groups		Independent sample *t*‐test			
	Stimulation group (*n* = 15) (Mean ± SD)	Sham group (*n* = 15) (Mean ± SD)	Statistic	Cohen's d effect size	Mean difference	*p*
**Age**	23,13 ± 3,60	22,93 ± 2,18	0.184	0.067	0.200	0,855
**Gender (F/M)**	9/6	10/5	0.367	0.134	0.066	0,716
**Education year**	15,73 ± 1,83	15,46 ± 1,59	0.425	0.155	0.266	0,674
**MoCA**	28,93 ± 1,43	28,73 ± 1,33	0.395	0.506	0.200	0,696
**Correct responses for OP**	15,73 ± 4,18	15,40 ± 4,26	0.216	0.079	0.333	0.830
**Incorrect responses for OP**	11,00 ± 4,12	11,53 ± 4,36	−0.344	−0.125	−0.533	0.733
**Correct responses for RP**	17,13 ± 4,49	15,67 ± 4,40	0.904	0.330	1.467	0.374
**Incorrect responses for RP**	9,33 ± 4,17	11,13 ± 4,58	−1.126	−0.411	−1.800	0.270
**Correct responses for NP**	17,80 ± 4,59	20,60 ± 3,25	−1.930	−0.704	−2.800	0.064
**Incorrect responses for TP**	8,87 ± 4,63	6,40 ± 3,25	1.690	0.617	2.467	0.102

**Abbreviations**: F, female; M, male; MoCA, Montreal Cognitive Assessment Scale; *n*, subject number; NP, new pairs; OP, old pairs; RP, recombined pairs; SD, standard deviation.

### 3.2 EEG Results

#### 3.2.1 Spontaneous EEG Response

To assess the effect of ITF tACS elicited during the FAST encoding on spontaneous eyes‐open EEG, spontaneous EEG data were analyzed using ANOVA. The group factor (stimulation vs. sham) was included as a between‐subject factor, while time (pre‐ vs. post‐stimulation), location (8 electrode clusters: Frontal 1 (F3 & F4), Frontal 2 (F7 & F8), Central, Temporal, Temporo‐Parietal, Parietal 1 (P3 & P4), Parietal 2 (P7 & P8), Occipital), and hemisphere (left, right) were treated as within‐subject factors.

Power analysis in the theta band did not reveal any statistically significant differences between groups or across time points (pre‐ vs. post‐stimulation) (*p* ≥ 0.05).

#### 3.2.2 Effects of Individual Theta Frequency tACS on ITF During FAST Encoding

To evaluate the effect of ITF tACS during the FAST encoding, theta peak frequencies measured in the spontaneous eyes‐open condition were compared pre‐ and post‐stimulation and during the FAST encoding and recognition phases (correct responses for OP, RP, and NP stimuli). ANOVA revealed no significant differences in individual theta frequency (*p* ≥ 0.05)

In the stimulation group, the spontaneous theta peak frequency was 5.04 ± 0.91 Hz pre‐stimulation and 5.22 ± 0.93 Hz post‐stimulation. During the encoding task, theta peak frequency was 4.92 ± 0.87 Hz; during recognition, theta peak frequency was 5.20 ± 0.94 Hz for correct OP responses, 5.23 ± 1.02 Hz for correct RP responses, and 4.97 ± 0.87 Hz for correct NP responses.

In the sham group, spontaneous theta peak frequency was 5.21 ± 1.06 Hz pre‐stimulation and 5.43 ± 1.19 Hz post‐stimulation. During encoding, theta peak frequency was 5.20 ± 0.84 Hz; during recognition, it was 5.13 ± 0.92 Hz for OP, 4.87 ± 0.91 Hz for RP, and 5.22 ± 0.96 Hz for NP correct responses.

#### 3.2.3 Theta Frequency in 100–400 ms

For event‐related 100–400 ms theta responses, group (stimulation vs. sham) was included as a between‐subject factor, while condition (theta response for correct OP, RP, and NP trials), location (8 electrode clusters: Frontal 1 (F3 & F4), Frontal 2 (F7 & F8), Central, Temporal, Temporo‐Parietal, Parietal 1 (P3 & P4), Parietal 2 (P7 & P8), and Occipital), and hemisphere (left, right) were treated as within‐subject factors.

A significant Group × Condition × Hemisphere interaction was observed (*F* (1.92, 53.89) = 4.47, MSe = 1.97, *p* = 0.017, *η* ^2^ = 0.003). In the stimulation group, theta power was higher in the right hemisphere for correct OP and RP trials, whereas in the sham group, theta power was higher in the left hemisphere for correct OP, RP, and NP trials.

To examine differences between theta responses during encoding and each condition, a two‐condition mixed‐design repeated measures ANOVA was performed.

**FIGURE 2:**
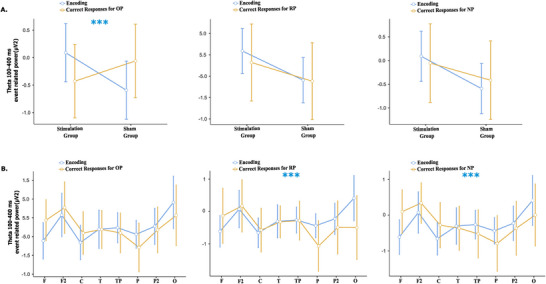
**Theta response results in 100–400 ms segmentation**. (A) Condition × Group Results. The X‐axis represents groups, and the Y‐axis represents event‐related theta power and (B) Condition × Location Results. The X‐axis represents locations, and the Y‐axis represents event‐related theta power (**Abbreviations**: C, central; F, frontal; F2, frontal (F7–F8); O, occipital; P, parietal; P2, parietal (P7–P8); T, temporal; TP, temporo‐parietal). ***shows the significant location for the question type (Blue: Encoding; Yellow: Correct Responses for OP, RP, and NP, respectively).

**FIGURE 3:**
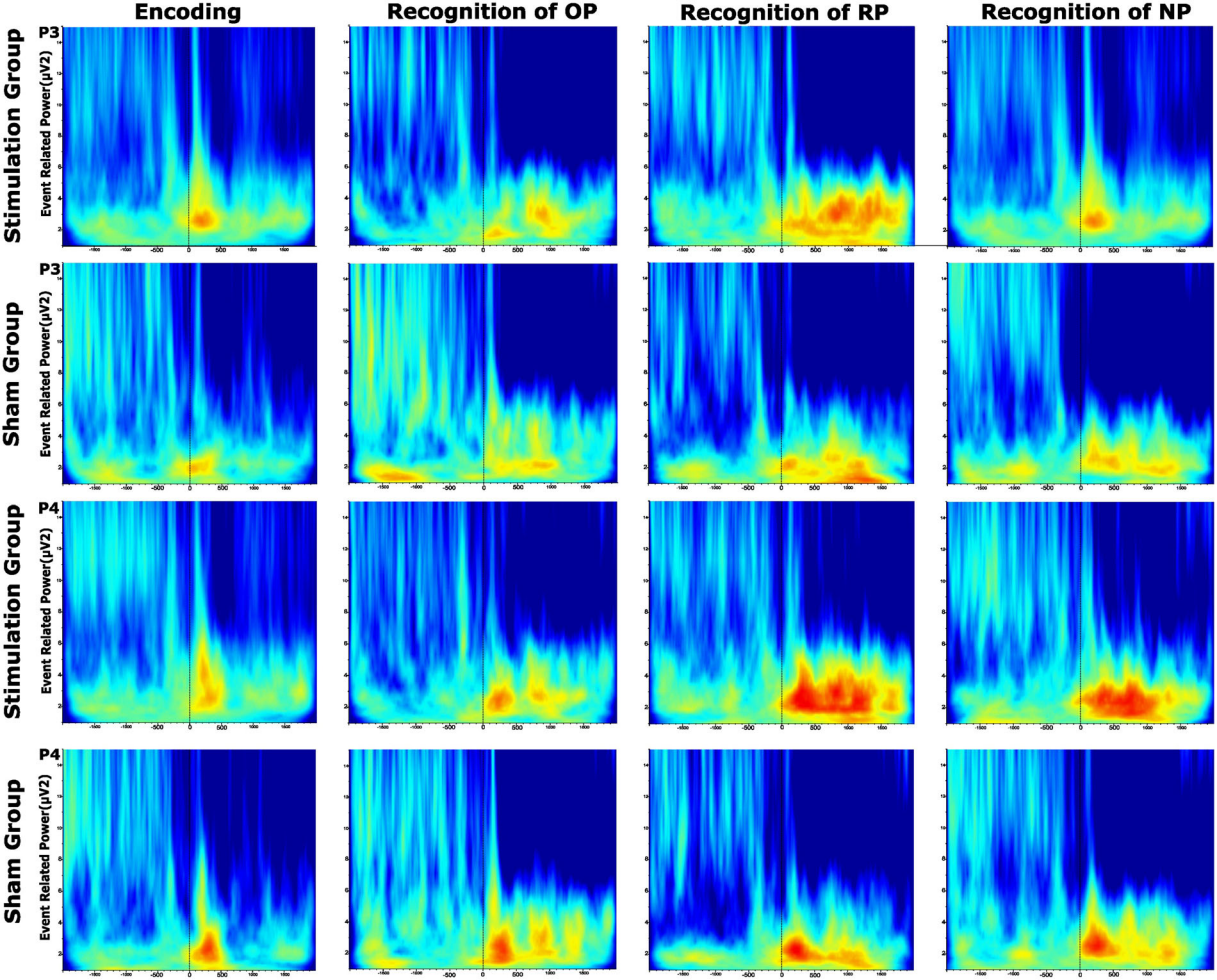
**The grand average of event‐related theta power analysis results in 100–400 ms** (The grand average figures of event‐related theta power analysis (4–7 Hz) in the time‐frequency domain for all conditions). The first and second rows illustrate the left parietal region for the stimulation and sham groups, respectively. The third and fourth rows depict the right parietal region for the stimulation and sham groups, respectively. The X‐axis represents time, and the Y‐axis represents frequency; when the picture first appears on the screen, it is marked as a zero point on the X‐axis. A 100–400 ms time interval is presented in the figure (**Abbreviations**: NP, new pairs; OP, old pairs; P3, left parietal; P4, right parietal; RP, recombined pairs).

Comparing theta responses during encoding with those observed for correct OP responses, a significant Condition × Group interaction was found (*F* (1.00, 28.00) = 10.19, MSe = 6.49, *p* = 0.003, *η* ^2^ = 0.267). In the stimulation group, theta power during encoding was higher than theta power during correct OP recognition, whereas in the sham group, theta power during encoding was lower than during correct OP recognition (Figure 2A, Figure 3).

When comparing theta responses observed during encoding with those observed during correct LP stimulus recognition, a significant Condition × Location interaction was found (*F* (3.26, 91.19) = 3.82, MSe = 3.28, *p* < 0.001, *η* ^2^ = 0.011). Theta power during encoding was higher than during RP recognition in temporal, temporo‐parietal, parietal (P3–P4, P7–P8), and occipital regions. Conversely, in frontal (F3–F4, F7–F8) and central regions, theta power was higher during RP recognition compared to encoding (Figure 2B, Figure 3).

Similarly, when comparing theta responses during encoding with those observed during correct NP stimulus recognition, a significant Condition*Location interaction was observed (*F* (3.46, 96.97) = 3.30, MSe = 2.87, *p* = 0.018, *η* ^2^ = 0.106). Theta power during encoding was higher than during NP recognition in temporal, temporo‐parietal, parietal (P3–P4, P7–P8), and occipital regions, whereas in frontal (F3–F4, F7–F8) and central regions, theta power was higher during NP recognition compared to encoding (Figure 2B, Figure 3).

## 4 Discussion

The aim of this study was to investigate the effect of individual theta‐frequency tACS stimulation applied over the left posterior parietal cortex during an associative memory encoding task, and to demonstrate the impact of modulating the left posterior parietal cortex on associative memory performance and theta frequency. For this purpose, stimulation and sham groups were formed and evaluated, which did not differ significantly in terms of demographic variables such as age and years of education, as well as individual theta frequency values measured during the resting state (eyes open) and the FAST task encoding phase.

Clouter et al., Herweg et al., and Kota et al. have demonstrated the contribution of the theta rhythm to the binding process through the formation of novel associations in memory, providing a theoretical foundation for theta‐frequency stimulation protocols (2017; 2020; 2020). The rationale behind theta‐frequency tACS is that externally applied rhythmic currents at the theta frequency can entrain intrinsic oscillatory activity within large‐scale functional brain networks, thereby enhancing theta synchronization and potentially improving memory performance (Ali et al. 2013; Hosseinian et al. 2021; Thut et al. 2011). Several studies have reported that theta‐tACS can facilitate associative memory function and may enhance performance in recognition tasks (Lang et al. 2019; Živanović et al. 2022). Although theta‐frequency tACS has generally been associated with improved memory performance, especially in associative memory, there is no consensus on the optimal stimulation frequency. The most commonly used range is 4–7 Hz, with 6 Hz frequently applied. However, Meng et al. (2021) challenged this approach by showing that 6 Hz theta‐tACS over the left posterior parietal cortex actually reduced associative memory performance. They suggested that fixed‐frequency stimulation may not align with participants’ endogenous theta rhythms, potentially causing phase mismatches or reduced synchronization and thereby impairing memory processes. In contrast, other studies have reported positive effects of fixed‐frequency theta‐tACS on memory, even when not targeting associative memory specifically (Shtoots et al. 2025; Debnath et al. 2025). These discrepancies highlight the need for further research to determine whether individualized or fixed‐frequency stimulation is more effective. To address this issue, recent work has explored individualized theta‐frequency tACS, where stimulation is tailored to each participant's peak theta frequency measured either at rest or during task performance. Evidence suggests that deriving the individual theta frequency from regions directly related to the cognitive function of interest is more effective than using global or unrelated electrode sites (Bjekić et al. 2022; Fröhlich and Riddle 2021; van Driel et al. 2015). This study represents the second investigation comparing tACS with a sham protocol using individual theta frequencies derived from EEG electrodes (C3, Cz, C4, P3, Pz, P4) associated with associative memory during an encoding task. While stimulation at task‐evoked individual theta frequencies has been reported to enhance associative memory (Živanović et al. 2022), in the present study, no significant differences were observed between the tACS and sham groups in the recognition task. Unlike Meng et al., no decrement in performance was observed, but stimulation did not produce a clear improvement either. Specifically, performance was similar between groups for OP responses; the tACS group showed better performance for RP responses, while for NP responses, the sham group outperformed the tACS group. Interestingly, these findings did not show a clear enhancement of associative memory following individualized theta‐frequency tACS. This null effect aligns with previous reports suggesting that theta‐tACS effects can be highly variable depending on task type, stimulation site, and individual differences in endogenous theta rhythms (Meng et al., 2021; Jones et al., 2019; Reinhart and Nguyen 2019).

These EEG findings are evaluated from several perspectives. First, no significant changes were observed in spontaneous theta activity. While tACS has the potential to modulate spontaneous EEG activity, this effect varies depending on stimulation parameters and the targeted brain region. tACS is a neuromodulation method designed to modulate brain oscillations by synchronizing them at specific frequencies. This indicates that tACS functions as an oscillatory entrainment tool rather than exerting direct excitatory or inhibitory effects. Accordingly, consistent with our findings, tACS appears to modify the timing and organization of neural activity rather than its amplitude (Elyamany et al., 2021; Ghiani et al., 2021; Ruhnau et al., 2016; Wu et al., 2021).

When examining the effect of individual theta‐frequency tACS on participants’ endogenous theta frequencies during the FAST encoding phase in EEG, no significant differences were observed. In previous studies, modulation was performed by applying ±1 Hz shifts to individual theta frequencies, and the behavioral outcomes of these modulations were evaluated (Aktürk et al. 2022; Herrmann et al. 2013; Ociepka et al. 2025; Paßmann et al. 2024; Roberts et al. 2018; Shtoots et al. 2024). In the present study, however, the theta frequency was individually determined based on electrodes prominent in memory during the encoding task and applied accordingly. Nonetheless, no significant difference was observed between the applied theta frequency and the theta frequency recorded during the recognition task. Unlike previous studies, no frequency shifting (±1 Hz) was performed, which may explain the absence of a marked effect. Additionally, no significant difference was observed in spontaneous EEG theta frequency. The literature suggests that individual theta frequencies determined from spontaneous EEG may not serve as a sufficient criterion for tACS applications (Meng et al. 2021). Although it remains an important research question whether theta frequency personalization should be based on spontaneous activity or task‐specific activity, these findings indicate that, at least in this context, calculating frequency from rest‐state EEG can yield similar results to task‐based determinations.

One EEG finding is a statistically significant interaction of Condition*Hemisphere*Group in theta responses between 100–400 ms. In the stimulation group, theta responses were higher in the right hemisphere for correctly identified OP and RP, whereas for NP, the response was higher in the left hemisphere. In contrast, in the sham group, theta responses were generally stronger in the right hemisphere across all condition types (OP, RP, and NP). The literature reports that hippocampal and temporal theta oscillations exhibit hemispheric differences, suggesting that left/right distinctions may play a critical role in memory functions (Miller et al. 2018; Penner et al. 2022). Increased theta power in the left hemisphere has been associated with hippocampal success, which implies that theta tACS may have influenced lateralization, thereby altering task performance (Miller et al. 2018). Notably, theta oscillations appear in brief and intermittent “bouts” that differ across hemispheres and are related to memory type and performance, although no definitive consensus has yet been reached (Penner et al. 2022). These findings support the notion that theta tACS can induce hemispheric‐level changes. Furthermore, the elevated theta response in the left hemisphere observed only for novel stimuli supports the left hemisphere‐specific role of the left parietal cortex in distinguishing new versus old information (Rutishauser et al. 2018; Petrovska et al. 2021). This observation aligns with previous literature suggesting that the left hemisphere is more engaged during familiarity processing, whereas the right hemisphere plays a more prominent role during retrieval.

The most important finding of the study is that, in the stimulation group, theta responses during encoding were higher than theta responses observed during recognition for OP, whereas in the sham group, theta power during encoding was lower than theta responses observed during recognition. Successful memory encoding appears to be associated with increased theta power in hippocampal regions, while theta power decreases during the successful retrieval of items. This finding suggests that theta tACS may enable improved performance with less cognitive effort, thereby enhancing cognitive efficiency (Aktürk et al. 2022).

The final EEG finding concerns the significant difference observed in the Condition*Location interaction. While no location‐related difference emerges during the recognition of OP images as in the encoding phase, regional differences are evident when RP or NP images are presented. During encoding, theta responses are stronger in the temporal, temporoparietal, parietal (P3–P4, P7–P8), and occipital regions, whereas recognition of RP or NP images elicits heightened responses in the frontal (F3–F4, F7–F8) and central regions. The hippocampus plays a central role in memory processes, particularly in binding distinct elements into a unified trace and in learning novel associations (Joensen et al. 2023). The temporal cortex contributes to the formation of semantic networks and associative processes, while the parietal cortex is critically involved in awareness, integration of information, encoding, familiarity–novelty discrimination, and retrieval strength (Jablonowski and Rose 2022). The increase in theta activity within frontal and central regions in response to NP or RP images is associated with monitoring, attention, and error‐control processes (Eisma et al. 2021). According to this framework, parietal areas are linked to memory‐initiated attentional processes (Cabeza et al. 2011; Jablonowski and Rose 2022). The role of parietal regions (P3, P4, P7, P8) in memory functions is also supported by previous studies demonstrating their role in associative binding. Theta rhythm is known to play a critical role in attention, memory, and cognitive functions (Başar et al. 2001; Khader et al. 2010; Sauseng et al. 2010). It is particularly emphasized in episodic memory encoding and retrieval, as well as in working memory tasks. In this study, theta responses observed in central (C3–C4) and frontal (F3–F4, F7–F8) regions during the recognition phase were significantly stronger than those during the encoding phase. The heightened theta response in these regions in the presence of RP images has been associated in the literature with the anterior attentional network and appears consistent with processes of target selection and inhibition (Kang et al., 2020).

The results of this study are also consistent with the Temporal–Frontal–Parietal Network Model, which posits that associative memory depends not on a single region but on the dynamic interaction among temporal, frontal, and parietal areas. While not definitive, the observed differences in responses to various stimulus types in our study point toward a possible involvement of parietal regions in attentional orientation and frontal regions in control mechanisms. Moreover, the binding effect of theta oscillations strengthens communication among these regions, thereby serving a critical mediating role in both encoding and recognition. Indeed, the increase in frontal theta activity during RP images, which facilitates accurate recognition, supports the notion that associative memory operates through a multi‐regional and oscillatory mechanism. When considered alongside these theoretical frameworks, these findings suggest that associative memory processes can be explained holistically at both the network and oscillatory levels.

Furthermore, these results might be interpreted within the framework of the Temporal–Frontal–Parietal Network Model. The network model posits that associative memory emerges through the interaction of the temporal lobe (hippocampus, parahippocampal regions), frontal control networks, and parietal attentional areas, mediated by mechanisms of timing, contextual integration, and attention (Ray et al. 2020; Shi et al. 2024). The enhancement of recognition performance by stimulation based on individual theta frequency during encoding suggests that temporal encoding mechanisms are later re‐engaged in frontal and parietal regions, thereby facilitating retrieval. This indicates that the “temporal synchronization” predicted by the network appears to be strengthened through external theta stimulation. The observation of right‐hemispheric theta dominance in the stimulation group and left‐hemispheric dominance in the sham group provides novel insights into network lateralization and supports the idea that the right hemisphere may play a more prominent role in episodic/detail‐oriented processes. The strong posterior theta (temporal–parietal–occipital) observed during encoding, together with the increased frontal/central theta during recognition, confirms the “dynamic regional transitions” predicted by the network model (Dave et al. 2022).

Although our study provides insights into the effects of individualized theta‐frequency tACS on associative memory, several limitations should be acknowledged. First, the sample size was relatively small, which may have reduced the sensitivity to detect small but meaningful effects. Second, while we applied standard analyses, more sophisticated or computationally intensive approaches (e.g., trial‐level mixed‐effects modeling across multiple electrodes, connectivity analyses) could further elucidate subtle neural effects. Stimulation was administered based on individual theta frequency derived from EEG; however, the inclusion of groups with fixed theta frequency or with ±1 Hz deviations could have revealed clearer distinctions in effects. Given the central role of the hippocampus, supporting these findings with high‐spatial‐resolution fMRI would have strengthened interpretability. Addressing these limitations in future studies, including recruiting larger participant cohorts and applying advanced analytical techniques, will likely improve the reliability and generalizability of the findings.

## 5 Conclusion

This study examined the effects of personalized tACS based on ITF on associative memory processes. Stimulation applied during encoding did not significantly enhance memory performance. No distinct theta effect was observed in spontaneous EEG during the eyes‐open resting state or in ITF measures. Time–frequency analyses revealed right‐hemispheric dominance in the stimulation group and left‐hemispheric dominance in the sham group within the 100–400 ms. Moreover, comparisons between encoding and recognition phases indicated that stimulation modulated the dynamics of theta responses, giving rise to hemispheric differences. Overall, although stimulation at an individualized theta frequency did not significantly enhance associative memory performance, it did modulate theta activity during the task. The most notable finding is that, in the stimulation group, theta power during encoding was higher than during recognition for OP stimuli, whereas in the sham group, theta power during encoding was lower than during recognition. This pattern suggests that successful memory encoding is associated with increased theta power in hippocampal regions, while theta power tends to decrease during item retrieval. These results indicate that theta tACS may facilitate cognitive processing by enhancing neural efficiency, potentially supporting improved performance with reduced cognitive effort.

## Author Contributions

**MsC Kahraman**: Conception, organization and execution of research project, acquisition and design, execution and critique of statistical analyses, writing of the first draft, review and critique the manuscript, tables and figures. **Dr. Saricaoglu**: Conception, organization and execution of research project, acquisition and design, execution and critique of statistical analyses, writing of the first draft, review and critique the manuscript, tables and figures. **Dr. Hanoglu**: Conception, organization and execution of research project, critique of statistical analyses, writing of the first draft, review and critique the manuscript.

## Funding

The authors have nothing to report.

## Conflicts of Interest

The authors declare no conflicts of interest.

## Ethics Statement

This study conducted with the approval of the Istanbul Medipol University Ethics Committee (Ethics Committee Report No: E‐10840098‐772.02‐6571).

## Consent

Participants provided written informed consent, and there was no compensation for participation as indicated in the written informed consent.

## Permission to Reproduce Material From Other Sources

The datasets used and analyzed during the current study are available from the corresponding author on reasonable request.

## Data Availability

The datasets used and analyzed during the current study are available from the corresponding author on reasonable request.
